# Immunological microenvironment and targeted therapeutics in multiple sclerosis: new insights in crosstalk between immune niches and CNS

**DOI:** 10.3389/fimmu.2025.1604987

**Published:** 2025-08-01

**Authors:** Xiaodi Sun, Feng Zhang, Luojinyun Wang, Gyeore Lee, Sibo Yang, Daqiang Zhou, Bohao Chang, Bo Hu, Yifan Zhou

**Affiliations:** Department of Neurology, Union Hospital, Tongji Medical College, Huazhong University of Science and Technology, Wuhan, China

**Keywords:** multiple sclerosis, autoimmune, neuroimmune, cerebral immune niches, targeted therapy

## Abstract

Multiple sclerosis (MS) is a chronic autoimmune disorder of the central nervous system (CNS) that predominantly affects young adults. However, current disease-modifying therapies demonstrate limited efficacy in addressing progressive disease subtypes, underscoring the urgent need for novel therapeutic strategies. Here, we systematically review the neuroimmune interactions underlying the pathogenesis of MS, with a focus on three key aspects: the immune niche, immune cell types, and cell-based therapies. We first discuss the evolution of brain-immune concepts, from early notions of immune privilege to modern understandings of brain-border immune niches (meninges, choroid plexus, and perivascular spaces). These compartments serve as critical interfaces where peripheral immune cells interact with CNS-resident immune cells. We then analyze the roles of specific immune cell subsets (e.g., T/B cells, myeloid cells and microglia) in disease progression, highlighting their functional heterogeneity across different MS subtypes. Furthermore, we highlight emerging MS immunotherapies-including chimeric antigen receptor (CAR) T regimens, mesenchymal stem cell interventions, microbiome modulation, and nanodelivery systems, which strategically target mechanistic nodes spanning neuroimmune niche regulation, inflammatory cascade blockade, and CNS neurorestorative capacities.

## 1 Introduction

Multiple sclerosis (MS) is a widespread chronic inflammatory disorder of the central nervous system (CNS), driven by immune-mediated processes and serving as the leading cause of acquired neurological disability among individuals aged 18 to 45 (1). Globally, approximately 2.5 million people globally are impacted by MS, with around 350,000 cases reported in the United States (2, 3). Most patients experience progressive neurological decline 10 to 15 years after onset. MS manifests in diverse clinical manifestations, encompassing clinically isolated syndrome (CIS), relapsing-remitting MS (RRMS), primary progressive MS (PPMS), and secondary progressive MS (SPMS). Notably, RRMS, the most common subtype, affects approximately 80% of patients, predominantly young women (4). Experimental autoimmune encephalomyelitis (EAE) model is widely regarded as the standard for investigating RRMS. Despite ongoing research, the exact cause and development of MS continue to be unclear, with contributions from both genetic predisposition and environmental influences suspected (5). The disease is marked by extensive demyelination, axonal degeneration, and astrogliosis in CNS white matter, leading to progressive neurological impairment (6).

The immune microenvironment within the central nervous system constitutes a sophisticated and dynamically regulated network. Brain border immune niches are emerging as pivotal anatomical compartments for investigating neuroimmune regulatory mechanisms. These particular niches function as entry points facilitating the migration of immune cells from the peripheral regions into the CNS, facilitating intricate crosstalk between immunocytes, cytokines, and membrane-bound receptors that orchestrates inflammatory modulation and tissue repair. Conventional multiple sclerosis research has historically underappreciated the mechanistic connection between brain border immune niches and disease pathogenesis. Emerging evidence demonstrates structural compromise of the blood-brain barrier (BBB), blood-cerebrospinal fluid barrier (BCSFB), and meningeal layers in both MS and EAE. These observations collectively indicate that dynamic remodeling of brain border immune niches is mechanistically intertwined with MS pathophysiology.

Activated antigen-presenting cells (APCs) and autoreactive T cells produce proinflammatory cytokines such as IFN-γ, TNF-α, IL-17, and IL-23, which promote cell-mediated immune reactions within the CNS. Conversely, Th2 cytokines (e.g., IL-4, IL-5, IL-10, TGF-β) exhibit protective effects. Dysfunctional regulatory T lymphocytes (Tregs), marked by Foxp3 expression, have played a role in the development of MS (7). Within the immunopathogenesis of neuroinflammatory conditions, IL-23 critically orchestrates the lineage commitment of lymphocytes toward pro-inflammatory effector profiles. This cytokine-mediated polarization is pathologically amplified in demyelinating autoimmune pathologies, with particular mechanistic relevance observed in CD4+ lymphocyte subsets demonstrating robust IL-17 synthesis capacity - a cellular population designated as Th17 lymphocytes (8). Recent evidence underscores the role of various immune cell subsets in the pathogenesis of MS, including peripherally derived and infiltrating T follicular helper (Tfh) cells (9), dendritic cells(DC) (10), NK cells, B cells (11, 12), and CNS-resident glial subsets (microglia (13), oligodendrocytes, astrocytes), which collectively orchestrate neuroinflammatory cascades. Methodological breakthroughs in high-resolution sequencing platforms, such as single-cell RNA sequencing (scRNA-seq) and single-nucleus RNA sequencing (snRNA-seq), have enabled multidimensional characterization of immune pathway crosstalk, cytokine-cell interplay, and proteome-wide interaction networks in MS, revolutionizing our understanding of spatiotemporal heterogeneity within disease-driving immune compartments. This review synthesizes contemporary advances in the pathogenesis of MS, alongside novel therapeutic targeting strategies, conceptualized through the framework of brain border immune niche.

## 2 Interactions between the immune system and the brain

Breakthroughs in high-throughput methodologies, such as flow cytometry and transcriptomic analysis, have dramatically expanded the multiplex capacity for concurrent measurement of immune parameters. This capability enables high-resolution quantitative profiling of neuroimmune responses within the CNS of individuals with MS. Leveraging these approaches, scientists have constructed analytical frameworks to investigate crosstalk between immune pathways, dynamic cytokine-cell interplay, and proteome-wide mapping of cellular interactions, advancing systematic characterization of neuroimmune networks in the human CNS. Consequently, high-throughput sequencing technology is increasingly utilized to elucidate pathogenic mechanisms in MS research (**Table 1**).

**Table 1:** Overview of single-cell sequencing studies in MS.

Methods and Technologies	Subjects of the study	Samples	Highlights	Refs.
scRNA-seq TCR-seq snRNA-seq	6 healthy individuals 5 RRMS patients	T cells in the blood and CSF Brain parenchyma	1. CSF T cells are largely distinct from the blood; 2. CSF T cells in healthy humans have an immunosurveillance function; 3. clonally expanded CSF T cells in MS patients show enhanced T cell activation and expression of cytotoxicity-related genes	(25)
CyTOF CITE-seq	61 monozygotic twins (one with MS, the other without)	PBMCs	1. MS signature in TH and myeloid cells; 2. CCR2–CSF2R are elevated in MS monocytes; 3. Dysregulation of the IL-2-CD25 axis	(26)
scRNA-Seq	4 MS patients 6 SCNI patients 4 noninflammatory controls 2 autoimmune encephalitis patients	CSF	1. Clonal expansions of T cells and plasmablasts; 2. Clonally expanded T cells resemble tissue-resident memory T cell phenotype; 3. CXCR6-CXCL16 axis in MS CSF: recruitment & maintenance of clonally expanded CD8+ T cells.	(27)
scRNA-seq TCR-seq	4 untreated MS 4 IIH controls	CD4+ T cells in the blood and CSF	1. revealed 12 distinct CD4+ helper T cell clusters; 2. CD4+ T cells from MS patients, HSP90B1, GNAI2, and S1PR1 expression were positively correlated with CNS migration, and ETS1 expression was negatively correlated.	(96)
scRNA-Seq scIg-Seq	16 RRMS 3 OND 3 HC 2 CIS	Blood and CSF	1. NF-κB and cholesterol biosynthetic pathways are activated in CSF memory B cells. 2. TGF-β1/SMAD signaling pathway was downregulated in CSF plasmoblasts/plasma cells. 3. Clonal expansion of IgM+ and IgG1+ CSF B cells with somatic hypermutation	(42)
CyTOF	11 MS patients 8 healthy controls	PBMCs	Significant expansion of T-bet+ B cells and CD206+ monocytes	(43)
BCR-seq	1 MS patient	Blood and CSF	During MS relapse, active inflammation-associated clonal IgA B cells can cross the BBB into the CSF.	(44)
CyTOF	14 MS patients 25 controls	Blood and CSF	In CSF from MS patients, B cell subsets expressing CD49d, CD45RA, CD20, high CD27, CD69, and CXCR3 were significantly increased.	(16)
scRNA-seq TCR-seq	7 untreated MS patients 7 controls	bone marrow (HSPCs and downstream lineages)	Increased differentiation of HSC to the medullary spectrum in MS	(22)
MARS-seq	EAE mice(acute phase day 16;chronic phase day 30)	CD45+CD11b+Ly6G+CD44hi cells in the inflamed CNS	Cxcl10 + monocytes are involved in CNS tissue damage	(106)
scRNA-seq	5 early active MS patients 1 healthy controls	CD45^+^ cells from the MS brains; healthy human microglia	The brain of MS patients is enriched in Hu-C2, C3, and C8 subpopulations, with high APOE, MAFB, and low core gene expression, unlike steady-state microglia.	(49)
scRNA-seq	2 RRMS patients 1 anti-MOG disorder patients	CSF cells and PBMCs	The myeloid cell population found in CSF is predominantly microglia	(50)
scRNA-seq	4 MS patients 4 IIH patients	CSF cells and PBMCs	Cytotoxic T helper cells increase in the CSF in MS	(48)
snRNA-seq	1 PPMS patients 9 SPMS patients 9 control patients	Frozen brain tissue (GM, WM, and meningeal tissue)	MS lesion progression is driven by a combination of selective neuronal damage and glial cell activation	(73)
snRNA-seq	5 progressive MS patients 3 controls	Brain (chronic active and chronic inactive MS lesions)	1. define microglia inflamed in MS (MIMS) and astrocytes inflamed in MS (AIMS); 2. C1q as a critical mediator of MIMS activation	(75)
spatial ISS scRNA-seq	EAE mice (before symptoms occurred, at symptom onset, peak, and late-stage) 4 progressive MS patients 2 controls	EAE mice (thoracic and cervical spinal cords and brains) Human (cervical spinal cord)	1. Active EAE lesions spread in a centrifugal manner; 2. dynamic induction and resolution of DA-glia	(77)
snRNA-seq snATAC-seq spatial transcriptomics	6 progressive MS patients 3 non-neurologic controls	Brain (NAWM, AL, CA, and RL)	1. MS-specific oligodendrocyte features influenced by the KLF/SP gene family 2. distinct cellular phenotypes around and within chronically active lesions; 3. B-cell co-expression networks	(78)
scRNA-seq	4 EAE mice 4 control mice	GFP+ cells from EAE-induced Pdgfra-H2B-GFP transgenic mice and Pdgfra-Cre-LoxP-GFP	OL and OPCs are not passive targets but instead active immunomodulators in MS.	(79)
scRNA-seq	EAE Mice (6 naive, 4 priming, 6 peak, 6 remission, 3 CFA) 4 MS patients,5 controls	Gfap+ astrocytes from mouse CNS (brain and spinal cord) Human (cortical and cerebellar)	MS-associated astrocyte subpopulations are characterized by decreased NRF2 activation and increased MAFG activation, accompanied by elevated DNA methylation, GM-CSF signaling activation, and enhanced pro-inflammatory pathways.	(84)
scRNA-seq RABID-seq	naïve *GfapTVA/G* mice EAE *GfapTVA/G* mice	*Gfap* ^+^ astrocytes	1. effectiveness of the RABID-seq technique;2. microglia expressing Sema4D and Ephrin-B3 control astrocyte responses through PlexinB2 and EphB3, respectively.	(93)

scRNA-seq, single cell RNA sequencing; snRNA-seq, single nucleus RNA sequencing; MARS-seq, massively parallel single cell RNA sequencing; snATAC-seq, Single-nucleus assay for transposase accessible chromatin by sequencing; RABID-seq, Rabies Barcode Interaction Detection followed by Sequencing; TCR-seq, T-cell receptor sequencing; BCR-seq, B-cell receptor sequencing; CyTOF, Cytometry by Time-Of-Flight; CITE-seq, Cellular Indexing of Transcriptomes and Epitopes by Sequencing; scIg-Seq, Single-Cell Immunoglobulin Sequencing; ISS, *In Situ* Sequencing; CIS, Clinically Isolated Syndrome; MS, Multiple Sclerosis; RRMS, Relapsing Remitting Multiple Sclerosis, Relapsing Remitting Multiple Sclerosis; PPMS, primary progressive multiple sclerosis; SPMS, secondary progressive multiple sclerosis; EAE, experimental autoimmune encephalomyelitis; CSF, Cerebrospinal fluid; PBMC, peripheral blood mononuclear cells; SCNI, subclinical neuroinflammation; IIH, idiopathic intracranial hypertension; OND, other neurological disease; HC, healthy control; HSPCs, hematopoietic stem and progenitor cells; HSC, hematopoietic stem cells; GM, grey matter; WM, white matter; NAWM:normal appearing white matter; AL, acute/active inflammatory demyelinating lesions; RL, repairing/remyelinating lesions; CA, chronic active lesions; MIMS, microglia inflamed in MS; AIMS, astrocytes inflamed in MS; OL, Oligodendrocyte; OPCs, Oligodendrocyte precursor cells.

### 2.1 Early perspectives and “immune privilege”

Conventionally, the brain has been considered an organ possessing “immune privilege”, where immune cell infiltration was believed to occur only during CNS inflammation and disease. Peripheral immune cell infiltration-induced focal inflammation holds a pivotal role in the neuropathological processes and development of MS. Nevertheless, recent research has unveiled a novel perspective, revealing that immune responses extend beyond their traditional role and actively participate in CNS maintenance and repair (14, 15). Discoveries have indicated that immune cells influence neurogenesis and cognition (16, 17).

### 2.2 Modern perspectives: immune niches in the brain

**Figure 1:**
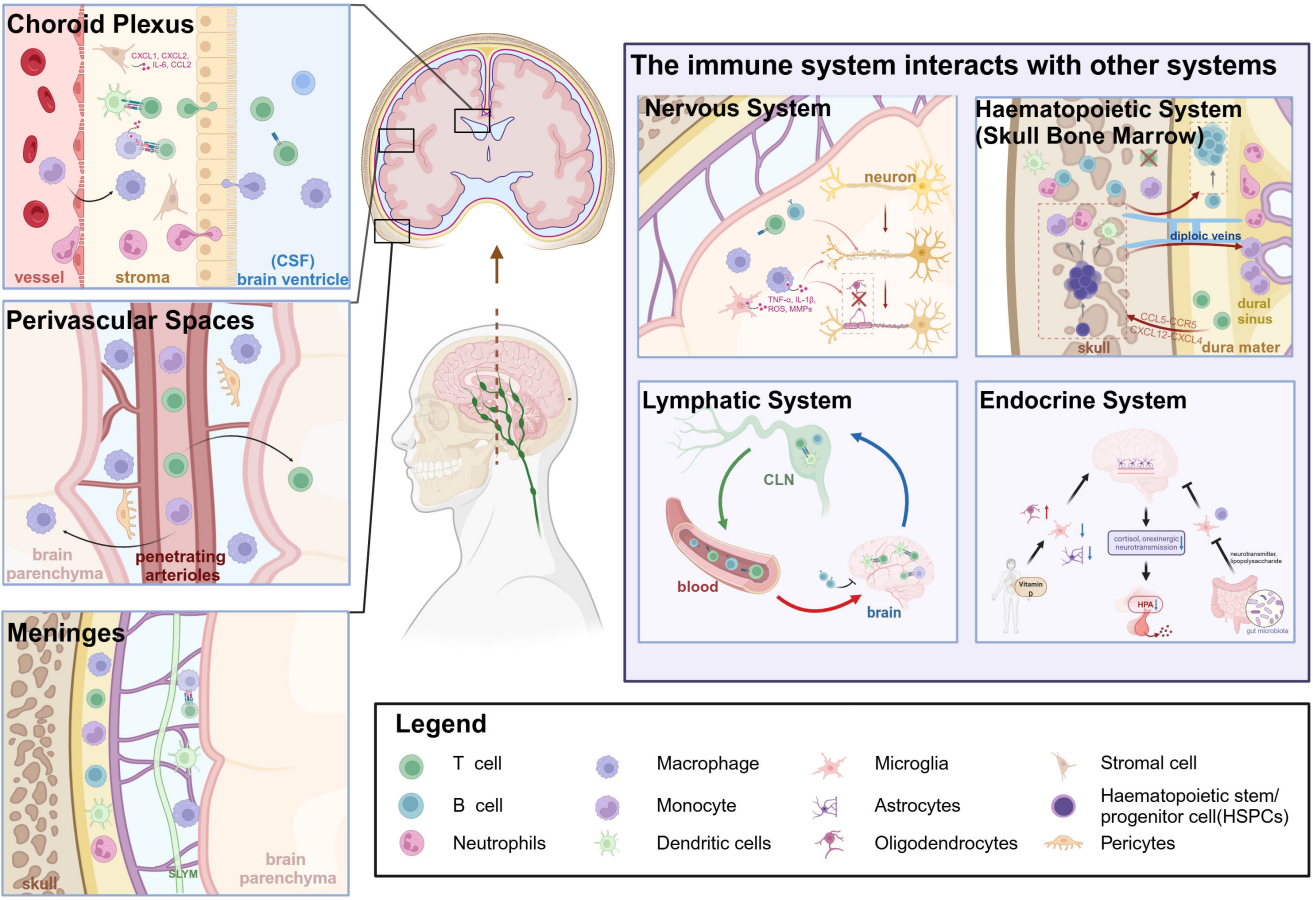
Overview of the pathogenesis of cerebral immune niches in Multiple Sclerosis and their interactions with other systems. Created in BioRender. Xiaodi, S. (2025) https://BioRender.com/a6k520m.

The anatomical boundaries of the brain include the BBB, BCSFB, and meninges (18). In this context, the BBB is considered the primary interface separating the periphery from the brain, with other structures serving the additional role of protective barriers that facilitate immune cell surveillance and defense functions. The notion of immune niches within the brain pertains to its border areas that support the presence and function of various immune cells, both innate and adaptive, thereby orchestrating brain function and repair (14, 19). These specialized immune niches include key areas like the choroid plexus (CP), perivascular spaces, and the meninges. Within these niches, peripheral immune cells infiltrate the brain parenchyma, inducing inflammation and cytokine release that modulate neuronal and glial cell activity and function, and orchestrate cross-system interactions (nervous, hematopoietic, lymphatic, and endocrine systems) to mediate disease pathogenesis (**Figure 1**). The meninges, divided into the inner pia mater, middle arachnoid membrane, and outer dura mater, each have distinct immune functions. In this regard, the dura mater is recognized as the fundamental immune ecological niche capable of sensing and presenting antigens, as well as releasing substantial quantities of cytokines (15). The subarachnoid lymphoid meninges (SLYM) within the subarachnoid space acts as a filtration mesh for material exchange, preventing the passage of CSF solutes exceeding 3 kDa in size (20). The choroid plexus, which houses a substantial population of immune cells, detects and responds to peripheral immune signals. The choroid plexus constitutes the BCSFB, serving as a pivotal location for immune cell infiltration and functioning as an essential brain barrier and immune hub (21). Moreover, perivascular spaces, including small penetrating blood vessels supplying blood to the brain and adjacent interstitial compartments, enable immune surveillance and response tailored to the brain’s immune requirements. In addition, the interconnected immune microenvironment encompassing immune niches also includes the skull bone marrow, offering a swift access route for immune cells (22), alongside the cervical lymph nodes (CLNs) responsible for draining the brain, which collect metabolic waste via the meningeal lymphatic vasculature (23). The existence and functional significance of these immune niches underscores the intricate interplay between the brain and the immune system, surpassing traditional views. Beyond their role in pathogen defense, these niches significantly impact neurogenesis, cognitive functions, and neural repair mechanisms.

### 2.3 Peripheral immune cells

Immune cells located in the periphery, along with their secreted mediators, possess the ability to penetrate into the brain parenchyma via specialized immune niches at the brain’s borders. These cells and factors play a crucial role in initiating and sustaining the pathological mechanisms underlying MS (24).

#### 2.3.1 T cells

The predominant presence of T cells within lesions is a crucial aspect of the pathogenesis of MS. Hafler’s team used a dual approach, integrating scRNA-seq with T-cell receptor sequencing (TCR-seq), to describe the enduring transcriptional signatures displayed by T-cells in the blood and CSF of healthy subjects (25). Their results showed that the T-cell populations in the CSF of healthy individuals comprised distinct clusters of memory CD4+ and CD8+ T cells and two major clusters: naive CD4+ T-cells and naive CD8+ T-cells in the blood. It is worth mentioning that the proliferation of these T cells was not extensively induced, and consequently did not trigger inflammatory cascades that are characteristically associated with MS, thereby underscoring their non-pathogenic nature within the context of healthy physiology. At the level of transcription, patients suffering from MS demonstrated heightened expression of genes involved in TCR recognition and the activation of T-cells, particularly within clonally expanded T-cell populations. Florian Ingelfinger et al. conducted an analysis on 61 sets of identical twins, where one twin was diagnosed with MS while the other, despite carrying the highest genetic predisposition, remained asymptomatic. The study found that the greatest difference in immune profiles between the twins was in cytokine receptors, which are the communication modes of immune cells (26). Furthermore, Beltrán et al. demonstrated the CSF of MS patients and subclinical neuroinflammation (SCNI) contained clonally expanded CD4+ T cells, CD8+ T cells, and B cells (27). In both MS and EAE, the migration of pathogenic CD4+ T cells into the CNS is the key event.

In the EAE model, we observed that the CD45+ immune cell subset, particularly CD4+ T cells, accumulates within the CP during the early stages of the disease progression and remains at high levels during the chronic phase, while the levels of these cells in the brain and spinal cord decrease (28). Chemokine receptor 6+(CCR6+) Th17 cells utilize the CP as an alternative pathway for accessing the CNS, thereby initiating EAE (29). Cytokines from Th17 cells, like IFN-γ and IL-17, stimulate the CP to release CCL20, facilitating the migration of pre-activated B cells and T cells into the CSF (30). The research conducted by Wu Zheng and colleagues revealed that knocking down CP-A2AR can inhibit the CCR6-CCL20 axis, thereby facilitating the reduction in the transit of Th17 cells across the CP into the brain parenchyma (21). Sabela discovered that in comparison to controls without neuroinflammation, patients with progressive MS exhibited a higher density of CD8+ T cells within the stroma of the choroid plexus. Furthermore, it was noted that MHCII+ antigen-presenting cells were frequently located in close vicinity to T cells, hinting at the CP involvement in immune surveillance within the CNS (31).

CD4+ T cells, also known as helper T (TH) cells, are crucial in orchestrating the immune response in MS (32). In the pathogenesis of MS, various subsets of Th cells exist, namely Th1, Th2, Th17, and Tregs, each fulfilling a unique role. Initially considered to be the primary causative cells of MS, Th1 cells secrete pro-inflammatory cytokines including IFN-γ and TNF-α (33, 34). Similarly, Th17 cells, distinguished by their secretion of IL-17, hold a significant role in MS progression (35). Elevated IL-17 levels in CSF and MS lesions correlate positively with disease severity. Tregs maintain immune tolerance and prevent autoimmunity, but in MS, their function and number are often impaired (36). It is now widely accepted that an imbalance between Treg cells and Teff cells within the CNS constitutes a novel pathophysiological mechanism in MS. Correction of this imbalance has been posited as a potentially efficacious therapeutic strategy. However, therapies targeting only T cells have proven ineffective in treating RRMS (37). Interestingly, within MS CNS lesions CD8+ cytotoxic T cells outnumber CD4+ helper T cells, highlighting their central role (38, 39).

#### 2.3.2 B cells

T lymphocytes have historically been regarded as central to the initiation and driving the progression of MS (40). However, due to the successful application of B cell depletion therapy specifically targeting CD20, the importance of B cells in MS immunopathogenesis has received increasing attention (11, 41). In the CSF of patients with MS, strong clonal expansion has been detected in the plasma cell population, with 90% of clones showing expansion, though 20% also show expansion in SCNI (27). In parallel, Ramesh et al. observed a 13-fold elevation in the number of B cells in the CSF of RRMS patients compared to healthy controls, accompanied by a skewed distribution of IgG1+ B cells favoring the CSF over the blood, and a balanced presence of IgG1+ and IgM+ B cells in the CSF. Notably, IgM-bearing cells were prevalent in naive and unswitched memory subsets, whereas IgG1-expressing cells were primarily restricted to plasma blast/plasma cells, switched memory B cells, and double-negative subsets (42). In the peripheral blood of early-stage MS patients there was an increase in B-cell subsets expressing CXCR3 (T-bet) and classic CD206+ monocyte subsets. Conversely, patients with aggressive MS exhibited an enrichment of B-cell subsets expressing CXCR3 (T-bet) (43). Furthermore, the presence of IgA-producing B cells (CD19+) within the inflamed CNS highlights the involvement of IgA-secreting cells in the pathogenesis of MS (44). Utilizing mass cytometry on both blood and CSF samples, Johansson and colleagues identified a novel subpopulation of small B-cells that is associated with MS. These cells exhibited a unique phenotype, including CD49d, CD69, CD27, CXCR3, and HLA-DR expression, which partially overlapped with memory B cells but did not fully match established B cell subsets, sharing similarities with plasma cells due to CD27 and CD20 expression, yet remaining distinct (16).

B cells located within the immune niches at the brain’s border primarily originate from two sources: the dura mater and the cranial bone marrow. The dura-associated lymphoid tissue (DALT) harbors germinal center B cells capable of differentiating into plasma cells upon antigenic challenge. These cells are adept at recognizing local antigens and can swiftly mount a humoral immune response (45). The cranial bone marrow functions as a direct reservoir for immune cells of the nervous system, with minute micro-osseous channels within the cranial marrow cavity linking to the trabecular veins of the meninges (46). B cells originating from the cranial bone marrow traverse microvascular channels within the cranial endothelium to migrate towards the dura mater, providing a continuous supply route of B cells to the CNS (47).

MS progression is influenced by the multifaceted functions of B-cells, acting as precursors to antibody-generating plasma cells. During inflammatory phases, these plasma cells release substances that have the capacity to cause demyelination in the CNS. Additionally, mature and memory B cells fulfill the function of APCs, releasing cytokines and chemokines. Notably, the involvement of pathogenic B cells may vary among different MS patient subgroups. Collectively, these results underscore the intricate nature and crucial importance of B-cells in MS, emphasizing the imperative for ongoing research into their functions and potential therapeutic applications.

#### 2.3.3 Macrophages and monocytes

Ingelfinger et al. discovered a monocyte subset in MS-affected twins characterized by high expression of CCR2 and granulocyte-macrophage colony-stimulating factor (GM-CSF) receptor-specific subunit CD116, accompanied by low CD14 expression and no CD16 expression (26). Shafflick et al. identified a monocyte subset termed “Mono2,” predominantly derived from cerebrospinal fluid. These Mono2 cells exhibit unique gene characteristics, including classical markers such as CD9, CD163, EGR1, and BTG2, alongside non-canonical markers like C1QA, C1QB, MAF, and CSF1R (also known as CD115). Furthermore, they express markers associated with vascular macrophages (LYVE1), microglia (including TREM2, TMEM119, and GPR34), as well as microglia resident at the CNS boundaries (STAB1 and CH25H) (48). The gene characteristics of Mono2 are similar to those of steady-state microglia described in previous studies (49, 50). Circulating monocytes can be classified into classical (phagocytic) monocytes, non-classical monocytes, and monocytes that display an intermediate transcriptional profile. Notably, both non-classical monocytes and those with an intermediate transcriptional profile characterized by high CD16 expression, possess patrol functions. Upon stimulation, they rapidly produce pro-inflammatory cytokines (51). In multiple sclerosis, increased circulating frequencies or frequencies in cerebrospinal fluid have been reported for these monocytes (52). Additionally, they produce heightened production of pro-inflammatory cytokines, notably including IL-6 and IL-12.

When monocytes enter tissues, they differentiate into tissue-infiltrating macrophages. Macrophages derived from the same monocyte lineage can display diverse functional profiles. M1 macrophages, characterized by their destructive nature, exhibit high levels of costimulatory molecules, cytokines that promote inflammation, and reactive oxygen species (ROS). These macrophages drive the differentiation of pro-inflammatory T cells, specifically Th1 and Th17 subsets. Macrophages that are alternatively activated, referred to as M2 macrophages, exhibit increased secretion of IL-10. These macrophages promote Th2 cell differentiation, display enhanced phagocytic abilities, and exhibit regulatory and homeostatic properties (51). In physiological states, the macrophages present within the CNS encompass parenchymal microglia and macrophages associated with the borders, referred to as border-associated macrophages (BAMs), together playing a crucial role in sustaining the homeostasis of the CNS (53). BAMs are composed of diverse macrophage subsets, such as choroid plexus macrophages, meningeal macrophages, and perivascular macrophages (54), with heterogeneous origins. For instance, some macrophages in the leptomeninges originate from progenitors in the yolk sac and fetal liver, enabling them to self-renew (55). In contrast, a subset of monocytes in the dura mater originates directly from adjacent skull bone marrow (56). Additionally, infiltrating monocyte-derived macrophages (MDMs) from the bone marrow hematopoietic system serve as another significant source of brain macrophages, such as those in the choroid plexus. Blood-derived macrophages are considered key contributors to the initiation of immunopathology in CNS diseases (53). A study has demonstrated that blocking the insulin-like growth factor-1 (IGF-1) signaling mechanism in BAMs results in a significant reduction of central nervous system inflammation in EAE mice (57). This finding validates the feasibility of targeting brain macrophages as a therapeutic strategy.

#### 2.3.4 Neutrophils

During inflammation, neutrophils enhance the permeability of the BBB, infiltrate leaky regions of the BBB and blood-spinal cord barrier (BSCB), and subsequently enter the CNS through compromised choroid plexus and leptomeninges, thereby exerting pathogenic effects (58). The choroid plexus epithelium synthesizes CXCL1 and CXCL2 chemokines, while the increased expression of adhesion molecules on its blood vessels of the choroid plexus facilitates the infiltration of blood-derived leukocytes into the brain parenchyma (31, 59). Neutrophils adhere via β2 integrins, specifically LFA-1 and Mac-1 (60), subsequently extravasate, and migrate into brain endothelium and other neural tissues. Additionally, they can obstruct blood flow by stalling in cerebral micro vessels, inducing ischemic phenomena (61). Neutrophils also release inflammatory mediators, such as ROS and cytotoxic granules, thereby exacerbating tissue injury (62). In murine models of cerebral ischemia, neutrophils release proteases and neutrophil extracellular traps (NETs), increasing BBB permeability and promoting inflammation and tissue injury (63). Despite the pathophysiological cascades through which neutrophils induce blood-spinal cord barrier dysfunction in multiple sclerosis remaining enigmatic, the decoding of their cellular choreography may establish novel therapeutic avenues targeting the neurovascular unit.

#### 2.3.5 Dendritic cells

DCs are highly efficient APCs that are essential to both innate and adaptive immune systems, capable of promoting or suppressing myelin antigen-specific immune responses (64). In CNS, DCs are localized within the choroid plexus, the meninges, as well as CSF. Their primary physiological role is to capture antigens that enter the CSF and conveying them to regional lymph nodes to activate antigen-specific responses (20). In EAE and MS, DCs exhibit both proinflammatory and immunosuppressive properties, which vary depending on the disease stage, course, and DC subtype. DCs are categorized into two primary subsets: conventional DCs (cDCs) and plasmacytoid DCs (pDCs) (65). Research indicates that DCs are major producers of IL-23, a cytokine with a pathogenic role in EAE and MS (66). An elevation in cDCs has been observed to exacerbate inflammation, whereas pDCs have been shown to foster the growth of Tregs, exerting an anti-inflammatory influence in EAE. A reduction in pDCs is associated with increased CNS inflammation and worsened clinical symptoms in EAE due to a reduced immune response involving Th1 and Th17 cells (67). Furthermore, both cDC and pDC numbers are significantly higher in the CSF of MS patients, in comparison to those with non-inflammatory conditions (68, 69).

David Schafflick and his colleagues employed scRNA-seq to study the cell atlas of CSF and blood, revealing the presence of previously unidentified myeloid dendritic cells (mDCs) in CSF. Their findings highlighted the increase in cellular heterogeneity in CSF and alterations in the transcriptional blueprint of blood cells in MS patients (48). Since mDCs are crucial for Treg differentiation, these results support earlier studies suggesting impaired Treg development in MS, potentially due to a deficiency of mDCs (70). In 2019, Böttcher et al. analyzed peripheral blood mononuclear cells from individuals at the onset of MS and found a marked decrease in CD141+ CD68low mDCs compared to healthy individuals (71). In MS, the transport, accumulation, and migration of DCs to the CNS are disrupted, contributing to disease pathology (72).

### 2.4 Resident immune cells in the CNS

#### 2.4.1 Microglia

Microglia are tissue-resident macrophages in the CNS that originate from the embryonic yolk sac. The heterogeneity of microglia has attracted significant attention, yet methods based on morphology, location, and surface markers cannot differentiate all subgroups. To tackle this issue, a study employed scRNA-seq to examine microglia (70). Researchers isolated cortical microglia from brains without CNS lesions and compared them to brains from early active MS cases. They identified four microglial clusters in the healthy control group, similar to clusters observed in previous mouse studies. Integrating scRNA-seq data from both MS and healthy brains, the study revealed seven microglial clusters: three exclusives to healthy brains, representing steady-state microglia; one group with cells from both MS and healthy brains, likely in a pre-activated state; and three clusters have been identified as being enriched or exclusive to MS brains. Of these, the unique ones exhibit reduced expression of core genes but heightened expression of APOE and MAFB. This diversity, influenced by spatial localization and the microenvironment, indicates that microglia play specific roles in MS pathology. Another study used single-nucleus RNA sequencing technology to analyze brain cells from MS lesions (73). Microglia from MS brains show activated phagocytic and amoeboid markers, confirmed *in vitro*. A separate study employing multimodal imaging techniques, which, while not isolating individual cells, analyzed cell morphology and location, distinguished blood-derived and tissue-resident microglia, differing in markers and morphology based on lesion type (74). This study highlighted the upregulation of C1q components in microglia bordering chronic active lesions, correlating with the presence of complement-related risk variants in patients with active lesions. Additionally, Martina Absinta et al. used scRNA-seq to investigate active lesion regions as well as healthy white matter. They identified two cell clusters specific to the edge of lesions: microglia active in MS inflammation (MIMS) and astrocytes (AIMS) (75). Beth Stevens and her team further identified nine microglial subgroups with distinct transcription patterns, each exhibiting unique gene expression profiles (76). This extensive analysis highlights the intricate roles played by MS and their significant contribution to the disease’s pathophysiology.

#### 2.4.2 Oligodendrocytes

While oligodendrocytes were previously considered solely as victims of immune cell attacks, Kukanja P’s study showed that oligodendrocytes are active not only in the peripheral zones of lesions but also throughout the spinal cord and brain (77). This raises the question of whether they are suppressed or potentially driving the disease in MS. Elkjaer ML et al.’s single-cell multi-omics study demonstrated that the KLF/SP gene family exerts a substantial influence on oligodendrocyte genetics in progressive MS, potentially through the mechanism of autocrine iron uptake signaling (78). Furthermore, these oligodendrocytes displayed inflammatory characteristics and were present both at the periphery and within chronically active lesions. Similarly, Falcão AM et al. discovered that oligodendrocytes and their progenitor cells in a murine model of MS possess immune cell properties, suggesting they are not only targets of immune attacks but also actively interact with immune cells, which contributes to disease regulation and progression (79). Additionally, oligodendrocyte precursor cells play a role in removing damaged myelin. Genes associated with MS susceptibility are active in both oligodendrocytes and their progenitor cells. These discoveries underscore the promising potential of oligodendrocytes as therapeutic targets, offering novel pathways for the development of future MS treatments and emphasizing their critical role in the disease.

#### 2.4.3 Astrocytes

Astrocytes are a crucial and highly heterogeneous component of the CNS (80). Astrocytes play a pivotal role in the development of lesions, enabling the infiltration of peripheral immune cells into the CNS (81, 82). By utilizing spatial transcriptomics and *in situ* hybridization methods, reactive astrocytes induced by inflammation are localized to distinct brain areas (83). Additionally, Francisco J. Quintana analyzed cells from the CNS of animals in an EAE model using Drop-seq technology, discovering multiple transcriptionally distinct subgroups of astrocytes. Among these, the Cluster 4 subgroup showed the greatest expansion during EAE induction. Further analysis revealed that within the Cluster4 subgroup, the expression of Nfe2l2, which encodes the transcription factor NRF2, was low. Conversely, the Cluster5 subgroup exhibited upregulated MAFG expression, which, in coordination with MAT2α, augments DNA methylation, also suppress transcription processes related to antioxidant and anti-inflammatory responses (84). These findings underscore the complex and dynamic roles of astrocytes in the pathology of MS and highlight potential as targets for therapeutic strategies.

## 3 Immune regulatory networks

The immune system comprises a sophisticated network consisting of a vast array of cells, receptors, and secreted factors. To be effective, immune responses require the seamless integration and orchestration of these diverse elements. To predict immune interaction outcomes and manage immune responses precisely, it is essential to study immune functions and dysfunctions at the pathway level rather than focusing on individual components. Understanding these underlying interaction networks is important for the development of precision therapies.

### 3.1 The disease-relevant immune cell crosstalk between the brain immune niche and the CNS

Interactions between resident immune-related cells and peripherally infiltrated immune cells can be observed in the brain. The expression of MHC molecules on APCs is indispensable for the recognition of antigens by TCRs. Antigen-presenting compartments in the CNS are constituted by both circulating mononuclear phagocytes of hematopoietic origin and tissue-embedded microglia, which are specialized myeloid sentinels endowed with CNS-specific maintenance functions. The co-stimulatory molecules they provide can effectively facilitate T-cell activation or suppression. For example, IL-12 and IL-6 play a crucial role in inducing the differentiation of Th1 and Th17 cells towards a pro-inflammatory state. Conversely, IL-10 fosters the development of Th2 cells, which exhibit an anti-inflammatory phenotype (85). Conversely, differentiated T-cells can also modulate the phenotypic conversion of APCs.

Follicular Th cells residing in lymphoid tissues secrete high levels of IL-21, which stimulates B-cell growth and differentiation into plasma cells, and potentiates follicular B-cell and Th17 responses, thereby exacerbating neuroinflammation (86, 87). Effector molecules, including granzyme B and perforin, are highly expressed by CD8+ T cells and have the capability to directly trigger the death of oligodendrocytes and neurons (88). Cytokines including TNF-α, lymphotoxin-α, and IL-6, which are secreted by B cells, can augment the proliferation of Th1 and Th17 cells, while anti-CD20 therapy targeting B cells can suppress this pathological reaction (89). B cells, especially memory B cells, also secrete GM-CSF, inducing the activation of pro-inflammatory myeloid cells (90). The upregulation of the CD22 protein, a B-cell receptor functioning as an inhibitory modulator of phagocytosis in microglia of aged individuals, may be correlated with decreased cognitive function in aged mice (90).

It has been demonstrated that by regulating microglial activation and the behavior of other non-neuronal cells, adaptive immune cells can exert indirect influences on synaptic processes and neuronal functioning. For instance, the presence of CD4+ T cells is essential for the progression and maturation of microglia (91). B cells infiltrate the brain during early mouse development and facilitate the proliferation of oligodendrocyte precursor cells via immunoglobulin M-Fcα/μR signal transduction, thus promoting myelin formation (92). Specifically, after demyelinating injury occurs in mice, myeloid cells with proinflammatory properties demonstrate a more aggressive role in myelin repair. MyD88-dependent signaling triggers these proinflammatory microglia to secrete TNFα, thereby promoting the generation of oligodendrocytes capable of new myelin formation (85).Francisco J. Quintana validated a novel single-cell sequencing technology called RABID-seq, which uncovered communication between microglia and astrocytes involving the EphB3-Ephrin-B3 and Sema4D-PlexinB2 signaling pathways (93). In chronic MS and its animal models, NK cells accumulate in the subventricular zone, particularly near neural stem cells that generate IL-15 and maintain NK cell function (94).

### 3.2 Immune cells cross the BBB

The BBB serves as a vital shield, safeguarding the central nervous system from external deleterious substances. It is comprised mainly consists of endothelial cells, pericytes with their basement membrane, astrocytes, and perivascular macrophages (95). In the context of MS, the interplay between immune cells and endothelial cells, along with the BBB, serves as a pivotal factor in the advancement of the disease. Studies have shown that immune cells, such as T and B cells, have the ability to cross the BBB, penetrate into the CNS, and trigger an inflammatory response through specific molecular pathways. These intricate mechanisms encompass the upregulation of cell adhesion molecules, the secretion of chemokines, and modifications in BBB permeability. Kendirli A et al. tracked the migration of CD4+ T cells from blood to CSF using scRNA-seq and TCR-seq techniques. They demonstrated that the expression of the migration-promoting genes *HSP90B1*, *GNAI2*, and *S1PR1* was positively correlated with their ability to migrate to the CNS, whereas the negative regulator *ETS1* exhibited a negative correlation with the migratory ability of CD4+ T cells from MS patients (96). IFN-γ and TLR9-activated T-bet-high IgG1 B cells may potentiate their recruitment via CXCR3 and amplify local immune responses within the CNS of MS patients (97). In comparison to healthy controls, brain microvascular endothelial cells sourced from individuals with MS exhibited compromised junctional integrity, reduced barrier functions, and decreased efflux pump activity. Furthermore, these cells demonstrated an inflammatory profile, characterized by elevated levels of adhesion molecule expression and heightened interaction with immune cells. Activation of Wnt/β-catenin signaling in endothelial progenitor cells isolated from MS was found to enhance barrier characteristics and attenuate the inflammatory response. Post-mortem brain tissue analysis of patients with MS revealed a reduction or disruption of proteins integral to tight and adhesion junctions, such as occludin, claudin-5, and vascular endothelial cadherin. Notably, extravascular leakage of serum components like IgG and fibrinogen was observed in the damaged BBB. Additionally, the crucial efflux pump P-glycoprotein (P-gp) was impaired within the lesions of MS patients. During MS, the endothelial cells of the BBB underwent an immunophenotypic shift, with the upregulation of intercellular adhesion molecule-1 (ICAM-1), vascular cell adhesion molecule-1 (VCAM-1), and atypical chemokine receptor 1 (ACKR1), thereby facilitating increased immune cell infiltration into the CNS (98).

Within the choroid plexus stroma, stromal cells release pro-inflammatory mediators, including IL-6, CCL2, CXCL1, and CXCL2 (99), in response to IL-1β produced by infiltrating activated APCs. Stimulated by cytokines such as IFN-γ and IL-17, the CP epithelium upregulates specialized trafficking molecules and secretes the chemokine ligand CCL20, thereby facilitating B and T cell migration into the cerebrospinal fluid (30).

Conversely, strategies aimed at inhibiting lymphocyte entry into the CNS, either by adhering to or sequestering lymphocytes in primary lymphoid organs, have proven effective in treating both MS and EAE. Adhesion blockade can be achieved with natalizumab, while lymphocyte sequestration can be induced using sphingosine-1-phosphate (S1P) receptor modulators such as siponimod and ozanimod.

### 3.3 The immune system interacts with other systems

The pathogenesis of MS transcends classical immune dysregulation, involving coordinated multiorgan crosstalk among the neurovascular unit, hypothalamic-pituitary-adrenal axis, and bone marrow-derived myeloid effector circuits.

The CNS constitutes the principal target of immunopathological assault in MS, with cardinal pathomechanisms centered on demyelination, axonal degeneration, and neurodegenerative processes. It has been shown that migratory Th cells from patients with MS express cytokines that target CNS homing molecules, thereby potentially fostering the proliferation of pathogenic CD4+ effector memory T cells within the inflamed CNS. TRM cells have been implicated in increased myeloid differentiation in MS (26). TRM cells are also recognized for their role in surveying the brain parenchyma in the absence of inflammation. It is postulated that CD8+ T-cell clones exhibiting a TRM phenotype, which are expanded, play a pivotal role in CNS injury. These cells constitute the primary lymphocyte population that persists and is shared between the CNS and CSF (27). Moreover, we identified a correlation between the expansion of clonal B-cells and gadolinium enhancement observed on MRI, hinting that the proliferation of clonal B-cells in the CSF becomes more pronounced during active demyelination and disruption of the BBB. This discovery extends a recent research that associated a heightened IgG index with elevated levels of neurofilament light chains in CSF, which serves as an indicator of axonal damage (100). The elevated somatic hypermutation (SHM) observed in IgM+ cells within CSF and the presence of a greater proportion of basic residues in the heavy chain complementarity-determining region 3 (H-CDR3) align with the exposure to neoantigens in the CSF. Furthermore, IgG1+ B cells exhibit longer H-CDR3 sequences compared to their counterparts in the blood, providing additional evidence of neoantigen exposure within the CNS (42).

Hypothalamic-pituitary-adrenal axis dysfunction is frequently observed in MS, which researchers suggest may be associated with orexinergic neurotransmission disturbances and abnormal cortisol secretion (101). Vitamin D plays a crucial role in regulating the immune system in MS by exerting anti-inflammatory and potential neuroprotective effects. It aids in myelin regeneration through oligodendrocyte precursor cells (OPCs), inhibits the activation of reactive astrocytes and M1 microglia to counteract neurodegenerative and oxidative stress processes, fosters the upregulation of neuroprotective factors, as well as regulates blood-brain barrier permeability (102). Alterations in the gut microbiota impact the susceptibility of mice in the EAE mice to neuroinflammatory demyelinating diseases. The gut-brain axis constitutes a sophisticated communication network encompassing the gut microbiota and the immune, nervous, and endocrine systems, whereby through neuroendocrine, neurotransmitter, and neuroimmune signaling mechanisms, the gut microbiota exerts an influence on the CNS (103), and can generate immunogenic endotoxins, such as lipopolysaccharide (LPS) (104), inducing autoimmune and neuroinflammatory responses within the brain. In MS, immune cells like microglia and meningeal NK cells become activated by antigens originating from the host or gut microbiota, leading to their migration into the CNS. This migration influences the formation and function of astrocytes (105), thereby promoting neuroinflammatory processes.

Through integrated single-cell transcriptomics and clonal lineage tracing, Liu et al. revealed pathological expansion of myeloid compartments in both MS patients and EAE models. Mechanistically, CNS-autoreactive T cells were shown to undergo CXCL12-CXCR4-mediated bone marrow homing, where they activate the CCL5-CCR5 chemotactic circuit to drive pathogenic myeloid expansion. Molecular profiling reveals dysregulated transcriptional networks in MS hematopoietic stem/progenitor cells (HSPCs), marked by enhanced granulocyte-monocyte progenitor (GMP) programs (AZU1+/MPO+/S100A8+) and hyperactivated monocyte-dendritic precursors (MDPs) with CST3hi/IFITM3hi/CTSShi signatures, along with upregulated myeloid-lineage transcription factors (CEBPZ↑, RARA↑, IRF8↑) that orchestrate pathological differentiation trajectories (22). Mildner et al. further distinguished eight monocyte subsets and three DC subsets in the acute and chronic phases of MS, highlighting the pathogenic Cxcl10+ and Saa3+ monocyte subsets originating from early myeloid progenitors (106). Collectively, these findings delineate a cell-autonomous bias of hematopoietic stem cells towards accelerated myeloid commitment in MS pathogenesis. The aberrant myelopoiesis drives excessive production of inflammatory neutrophils and monocytic effectors that breach the blood-brain barrier, creating self-reinforcing neuroinflammation through feedforward amplification of demyelinating cascades. Through the analysis of twin pairs, F. Ingelfinger and colleagues discovered that twins with MS exhibited a shift in bone marrow compartments, characterized by a transition from nonclassical monocytes to inflammatory classical monocytes, accompanied by a reduction in the type 1 interferon gene signature. These monocyte subpopulations demonstrated heightened expression of CCR2 and GM-CSF receptors, indicating their heightened sensitivity to inflammatory stimuli (26).

### 3.4 Immunological microenvironment comparison among different types of MS

MS is characterized by a dynamic disease continuum and its course must be viewed from a developmental perspective. In the developmental trajectory of MS, CIS represents the initial stage, suggesting that some patients are at risk of progressing to RRMS and that over time the majority of patients with RRMS will progress to SPMS. The presence of focal plaques is a central pathological characteristic that is common to all types of MS, which manifest pathologically as demyelination in the peripheral area of small post-capillary veins. However, these subtypes exhibit significant differences in their immunological microenvironments. Firstly, RRMS is characterized by intermittent disease attacks and remissions. The immunological microenvironment in RRMS shows periodic exacerbation and alleviation of inflammatory responses. scRNA-seq of lesion tissues in RRMS patients reveals numerous active inflammatory T cells, especially Th1 and Th17 cells, as well as abnormally activated B cells that produce autoantibodies. During remission periods, Tregs are enhanced, helping to suppress inflammation (107). Secondly, SPMS presents as a gradually worsening disability with relatively stable inflammatory reactions. In SPMS patients, single-cell sequencing shows fewer increases in immune cells but a significant decrease in neuroprotective Tregs. This reduction leads to ongoing inflammation and neuronal damage. The imbalance in the immunoregulatory network results in the loss of neuroprotective mechanisms, accelerating disease progression (42). Thirdly, in PPMS, the disease presents with an earlier onset of progressive disability, with no obvious intermittent inflammatory attacks. Lesion tissues in PPMS patients, analyzed using single-cell sequencing, reveal persistently activated inflammatory T cells, especially Th17 cells, and an abnormal increase in macrophages. Additionally, the function of Tregs is inhibited, leading to sustained autoimmune responses without effective control (108). In summary, RRMS is dominated by periodic inflammatory fluctuations and enhanced Treg roles. In contrast, SPMS and PPMS show sustained activation of inflammatory cells and an imbalance in the inflammatory regulatory network, contributing to progressive disease deterioration. Understanding these differences in the immunological microenvironments provides a theoretical basis for tailored treatment and intervention strategies targeting specific immune cell subtypes in different types of MS.

### 3.5 Immunological microenvironment comparison between MS and other autoimmune systemic diseases

MS is a complex disorder involving neurons, immune cells, and glial cells, marked by clinical and pathological heterogeneity. The MS microenvironment comprises various signals that regulate remyelination and myelin sheath disruption. Traditional bulk genomic and transcriptomic analyses have provided significant insights into disease remission and activity but may obscure critical signals from specific cell populations. Understanding these signals is crucial for improving MS treatment responses and advancing stem cell and immune cell therapies (42). Compared to other autoimmune systemic diseases, the immunological microenvironment of MS displays unique features. This group of cells primarily consists of T cells, B cells and macrophages, all of which play a role in the inflammatory process. In RRMS patients, single-cell sequencing has revealed numerous active inflammatory T cells, particularly Th1 and Th17 cells, along with abnormally activated B cells that produce autoantibodies (107). In other autoimmune diseases, like systemic lupus erythematosus (SLE) (100) and rheumatoid arthritis (RA)[82], the types of inflammatory cells involved may vary, with T cells, B cells, and macrophages playing more prominent roles. Additionally, the specificity of autoantibodies to target organs differs. In MS, B cells produce autoantibodies against self-antigens, affecting nervous system tissues. In contrast, SLE and RA involve autoantibodies targeting different tissue organs (109). Additionally, MS exhibits abnormal cytokine expression patterns, including pro-inflammatory cytokines like IL-17 and IFN-γ, as well as anti-inflammatory cytokines such as IL-10 and TGF-β. In other autoimmune diseases, the patterns and characteristics of cytokine expression may differ. In MS, the dysfunction of immunoregulatory cells, such as Treg cells, gives rise to a disruption in immune tolerance, ultimately causing ineffective control of autoimmune attacks. While immunoregulatory cells also play essential roles in other autoimmune diseases, their mechanisms of action may differ. In conclusion, significant differences exist in the immunological microenvironment between MS and other autoimmune systemic diseases, contributing to distinct pathological features and clinical manifestations. Understanding these differences is crucial for elucidating the pathogenesis and pathological processes of various autoimmune diseases, providing new perspectives and strategies for personalized treatment and intervention. However, additional research is necessary to fully understand the intricate mechanisms underlying these differences.

## 4 Targeted therapeutic strategies for immunomodulation

Inflammation plays a pivotal role in the early stages of MS, which is known as the “golden window” for treatment. During this phase, widespread neurological damage has not yet accumulated, and the body’s reparative mechanisms are still active, making Disease Modifying Therapies (DMTs) most effective. However, as the disease progresses, the capacity for repair diminishes, leading to irreversible disability. The clinical manifestations and treatment responses of MS vary extensively, thus presenting challenges for accurate diagnosis and effective therapy. Therefore, personalized molecular targeted therapy is urgently needed.

Since the introduction of IFN-β for the treatment of MS in 1993, a growing array of immunomodulatory medications has been shown to be effective and has gained widespread utilization. These drugs help reduce inflammatory brain damage and decrease the likelihood of clinical relapse through various mechanisms. Key therapeutic drugs include IFN β-1a, β-1b, and pegylated interferon β-1a, which reduce inflammatory responses; glatiramer acetate, which affects antigen presentation to T cells (110). Fingolimod, siponimod, ozanimod, and ponesimod, which regulate immune cell movement by affecting S1P receptors; dimethyl fumarate, which inhibits secretion from dendritic cells; and DNA synthesis inhibitors such as cladribine and teriflunomide (111). Additionally, some immunosuppressive agents, including azathioprine, cyclophosphamide, glucocorticoids, IVIG, and mitoxantrone, are used based on limited evidence.

**Figure 2:**
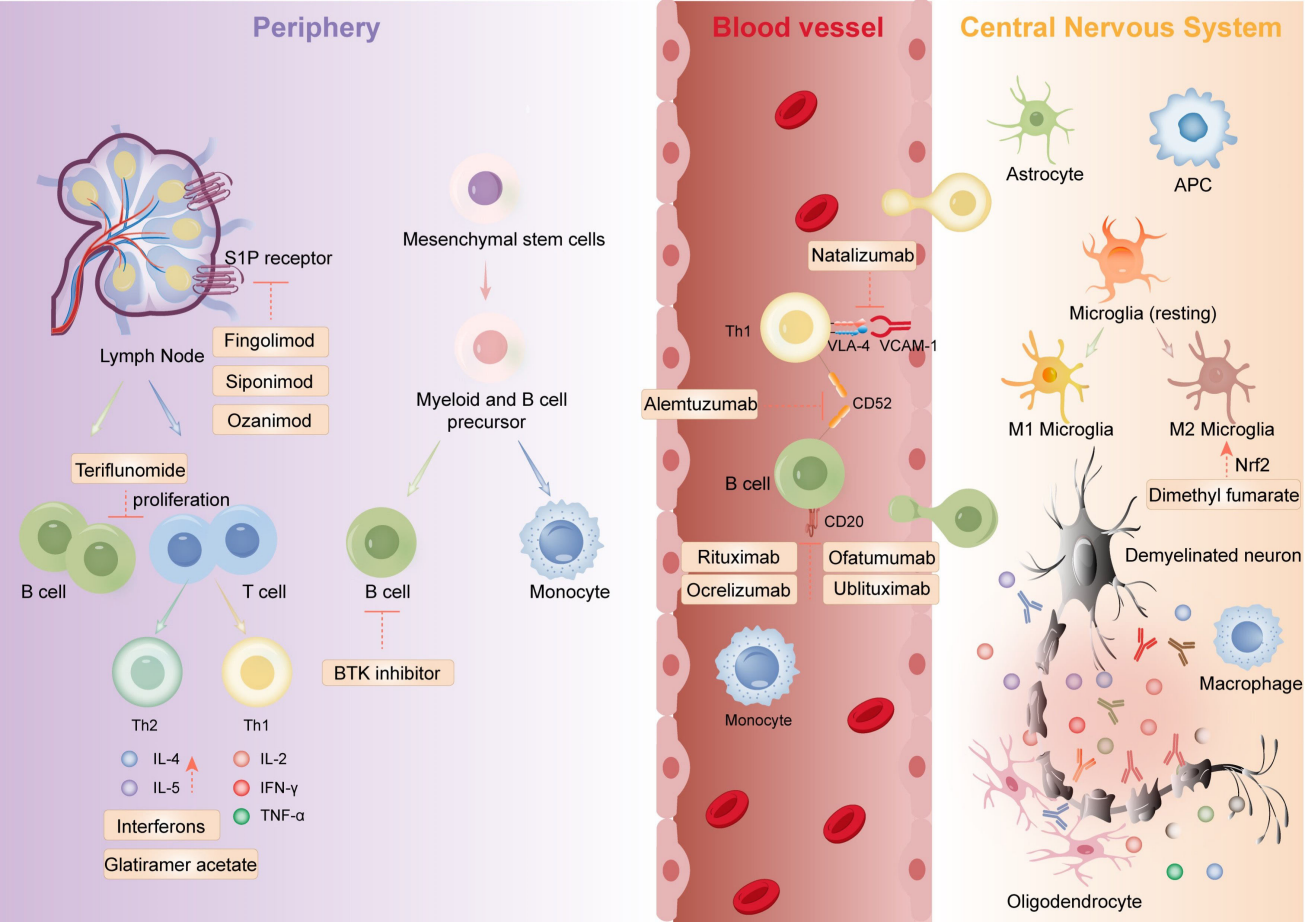
The mechanistic framework of marketed immune-targeting therapeutics for multiple sclerosis encompasses: orchestrating lymphocyte polarization, suppressing pathogenic Th17/Treg imbalance, enforcing CNS infiltration blockade via endothelial adhesion modulation, and stimulating oligodendrocyte-mediated remyelination. Figure created with Adobe Illustrator.

Compared to traditional treatments, monoclonal antibodies can more precisely target specific immune pathways or cells, reducing damage to healthy tissues and demonstrating better efficacy and safety in individual patients. For example, CD52 inhibitors such as alemtuzumab modulate the immune system by inhibiting the CD52 antigen, reducing inflammatory responses. Natalizumab prevents immune cells from entering the CNS by inhibiting integrin α4, thereby reducing inflammation. CD20 inhibitors, including ocrelizumab, ofatumumab, rituximab, and ublituximab, modulate the immune system by inhibiting the CD20 antigen, thus reducing inflammation. An overview of therapeutic targets for DMTs in MS is shown in **Figure 2**.

Advancements in single-cell sequencing technology have led to more precise targeted therapies. β-interferon confirmed immunomodulatory drugs’ efficacy in MS. Immunosuppressants like azathioprine and glucocorticosteroids have also been used. Since then, DMTs has shifted to targeted agents like glatiramer acetate, cladribine, and teriflunomide. Monoclonal antibodies, with their precision in targeting immune pathways, have emerged as key therapies, offering tailored efficacy and safety.

Furthermore, several preclinical and clinical trial drugs are being developed for MS treatment, showing considerable efficacy in experimental animal models (**Table 2**). For instance, in EAE models, oral administration of adrenocorticotropic hormone (ACTH) reduces IL-17 and IFN-γ in the CNS (112). By blocking the Dihydroorotate Dehydrogenase (DHODH) enzyme, Calcium vidofludimus (IMU-838; Immunic AG, Germany) inhibits the intracellular metabolic processes of activated T and B cells (113). Repeated immunization with all-trans retinoic acid (ATRA) and CAF16 liposomal adjuvant in an EAE model resulted in T-cell phenotypic transformation (114). Methyltransferase Setd2, an epigenetic regulator, inhibits the development of Th17 cells while promoting iTreg cell polarization through phospholipid remodeling (115). B-cell activation factor(BAFF) shifts B-cell pools towards a regulated phenotype, affecting B-cell function (116). Fasudil inhibits microglia-mediated neuroinflammation (117, 118). Frexalimab blocks the co-stimulatory CD40/CD40L cell pathway (119). Immune Cell Tolerance Therapy vaccines induce tolerance in the immune system by injecting specific antigens, suppressing autoimmune responses, and reducing disease symptoms (120). There are several factors to consider when choosing and combining medicines, including the patient’s medical condition, the severity of the disease, and the patient’s tolerance to medicines. The comprehensive use of different drug categories can effectively control MS symptoms and progression.

**Table 2:** Clinical trials of drugs for the treatment of MS.

Clinical trial	Name of the drug	Type	Refs.
Phase III	Calcium Vidofludimus	Interrupts the mitochondrial enzyme involved in *de novo* pyrimidine synthesis of DHODH	(113)
Phase II	all-trans retinoic acid (ATRA)	T-cell phenotypic transformation	(114)
Phase II	methyltransferase Setd2	T-cell phenotypic transformation	(115)
Two double-blind randomized trials	Fasudil	Microglia phenotypic transformation	(117, 118)
Randomized double blind phase IIb trial	Frexalimab	Target CD40/CD40L	(119)

There are some preclinical drugs that have shown effectiveness in animal models of MS disease. The use of suppressor Tregs to induce durable tolerogenic autologous chimeric antigen receptor (CAR) T cell therapy targeting myelin oligodendrocyte glycoprotein (MOG), effectively modulates CD4+ pathogenic cell activity and diminishes microglia activation, thereby significantly reducing inflammation in the CNS of EAE mice (121). Programmed cell death 1 (PD-1) interacts with programmed cell death ligand 1(PD-L1) to maintain immune tolerance. Soluble PD-L1 (sPD-L1) alleviates clinical symptoms of MOG-induced EAE by inhibiting CD86, CCR7, and splenic DC transport (122). The microbiota-metabolite-immunity axis also has the potential to influence EAE disease progression. Ning and Li,Xinyan observed that oral inulin alters intestinal microbiota, reducing Th17 cells and inflammatory cytokines (123). Additionally, Bing and Han, Lin found that oral ellagic acid(EA) boosts short-chain fatty acid-producing bacteria(e.g., Alloprevotella), upregulating propionate(C3) and reducing the secretion of inflammatory cytokines by pathogenic Th17 cells (124). Autologous mesenchymal stem cell (MSC) therapy addresses both immune and neurodegenerative mechanisms in MS. Furthermore, MSC-derived neural progenitor cells (MSC-NPs) support myelin regeneration and have immunomodulatory properties (125). Extracellular vesicles (EVs) derived from MSCs, containing miR-181a-5p, have been shown to decrease STAT3 expression in T-cell regulation. These EVs also inhibit microglia inflammation and cellular death through the USP15-mediated RelA/NEK7 axis (126, 127). Furthermore, antigen-specific Tregs induced by adoptive cellular therapy (ACT) in treated mice resulted in a decrease in inflammatory infiltration and demyelination within the spinal cord (128). Nanomaterials are also used in MS therapy. The combination of nanoligomers (NF-κB1 + TNFR1, known as NI111, and NF-κB1 + NLRP3, known as NI112) reduce neuroinflammation without adverse effects on organoid function (129). Graphene oxide (GO) is biocompatible, but its immunomodulatory effect weakens when myeloid-derived suppressor cells (MDSCs) are exposed to rougher rGO (90), where cellular size correlates with apoptotic activation (130). Morris and Rad, among others, have devised a biomaterial scaffold intended to replace diseased tissue and establish engineered immunological niches for monitoring disease progression. This enables the recognition of pathological signals at the onset of disease and the prompt application of targeted immunotherapy (131, 132). **Table 3** presents a comparative analysis of promising investigational therapeutic strategies and clinically validated DMTs in MS management.

**Table 3:** A comparison of disease-modifying therapies (DMTs) with investigational approaches.

Therapy/Drug Name	Mechanism	Phase of development	Therapeutic goals	Refs.
Disease-modifying therapies (DMTs)				
Interferon-β (IFN-β-1a, IFN-β-1b, Peginterferon-β-1a)	Reduce inflammatory responses by modulating immune cell activation and cytokine production	FDA-approved (introduced 1993)	Approved for the treatment of RRMS; IFN-β-1b approved for RRMS and SPMS; Mitigate relapse risk and inflammatory CNS damage	(111)
Glatiramer Acetate	Induce immune tolerance by altering antigen presentation to T cells	FDA-approved	Approved for the treatment of RRMS and CIS; Modulate immune dysregulation and reduce inflammatory activity	(110)
Fingolimod/ Siponimod/ Ozanimod/ Ponesimod	Sequester lymphocytes in lymph nodes via S1P receptor modulation	FDA-approved	Fingolimod and Ozanimod approved for RRMS; Siponimod approved for SPMS; Ponesimod approved for RRMS and SPMS; Limit immune cell infiltration into the CNS and reduce neuroinflammation	(111)
Dimethyl fumarate	Inhibit secretion from dendritic cells and activate various cytoprotective signaling pathways	FDA-approved	Approved for the treatment of RRMS; Reduce oxidative stress and inflammatory demyelination	(111)
Teriflunomide	Selectively deplete lymphocytes through DNA synthesis inhibition by inhibiting DHODH	FDA-approved	Approved for the treatment of RRMS; Lower annualized relapse rates and disability progression	(111)
Cladribine	Consume adaptive immune cells by interfering with DNA synthesis and repair	FDA-approved	Approved for the treatment of RRMS; Reduce the relapse rate, MRI activity and the risk of disability progression	(111)
Alemtuzumab	Deplete circulating B/T lymphocytes, inducing immune reconstitution by inhibiting the CD52 antigen	FDA-approved	Approved for the treatment of RRMS; Induce prolonged disease remission (requires strict safety monitoring)	(111)
Natalizumab	Prevent immune cells transmigration across the blood-brain barrier by inhibiting integrin α4	FDA-approved	Approved for the treatment of RRMS; Suppress CNS inflammation (PML risk monitoring required)	(111)
Ocrelizumab/ Ofatumumab/ Rituximab/ Ublituximab	Deplete B cells, reducing antibody production and proinflammatory cytokine secretion by inhibiting the CD20 antigen	FDA-approved	Approved for the treatment of RRMS; Ocrelizumab approved for PPMS; Reduce relapse rates and slow disability accumulation	(111)
Immunosuppressants (Azathioprine, Cyclophosphamide, Mitoxantrone)	Non-selectively suppress immune cell proliferation	FDA-approved (limited evidence)	Control acute exacerbations (short-term use due to toxicity risks)	(111)
Investigational Approaches				
CAR T cell therapy	Target MOG-specific suppressive Tregs to modulate CD4+ pathogenic T-cell activity and microglia activation	Preclinical	Delay the onset of EAE mice and reduce the activity of immune cells	(133)
Soluble PD-L1	inhibit CD86, CCR7, and splenic DC transport	Preclinical	Reduce the severity of limb disability in EAE mice	(122)
MSC-NP therapy	Support myelin regeneration and have immunomodulatory properties	Investigational/Preclinical	Improve clinical symptoms and neurodegeneration	(125)
Extracellular vesicles	MSC-EVs containing miR-181a-5p suppress T-cell STAT3 signaling and microglia inflammation via USP15-RelA/NEK7 axis	Preclinical	Reduce neuroinflammation in EAE mice and alleviate clinical symptoms	(127)
antigen-specific Tregs induced by adoptive cellular therapy (ACT)	Induce immune tolerance, reducing spinal cord inflammatory infiltrates	Preclinical	Decrease disease severity scores and reduce inflammatory infiltration and demyelinate in the spinal cord	(128)
Tolerogenic Nanovaccine	Induce tolerance in the immune system by injecting specific antigens	Preclinical	The symptoms of EAE mice were alleviated	(120)
oral inulin	Alter intestinal microbiota infecting the microbiota-metabolite-immunity axis, generating butyric acid to reduce Th17 cells and inflammatory cytokines	Preclinical	ameliorated the severity EAE in mice with reductions in inflammatory cell infiltration and demyelination in the CNS	(123)
oral ellagic acid	Boost short-chain fatty acid-producing bacteria upregulating propionate(C3) and reducing the secretion of inflammatory cytokines	Preclinical	Inhibit polarization of Th17 cells, ultimately hindering the development of EAE	(124)
Nanomaterials	NF-κB1/TNFR1 nanoligomers (NI111) and NF-κB1/NLRP3 nanoligomers (NI112) selective inhibit inflammasome and preserve key components of immune function; Graphene oxide (GO) weakens the immune-modulatory effect of myeloid-derived suppressor cells (MDSCs)	Preclinical	reduce neuroinflammation without any observable negative impact on organoid function	(129, 130)
Biomaterial Scaffolds	Engineered niches for real-time disease monitoring and targeted immunotherapy delivery	Investigational	Enable early intervention and personalized treatment strategies	(131)

## 5 Conclusion and future perspectives

The CNS was traditionally viewed as immune-privileged, with mechanisms in place to limit immune responses and prevent potential neurological damage. However, emerging research has revealed that the CNS and immune system are interconnected more intricately and dynamically. Beyond the resident immune cells of the CNS, specifically microglia, which are responsible for local immunosurveillance and regulation, there is a broader cerebral immune network involving multiple peripheral immune components. This network includes the meninges, the skull’s bone marrow, the choroid plexus, and the lymphatic drainage system, all of which contribute to neuroprotection, antigen presentation, and immune regulation. However, In MS, this immune microenvironment is disrupted by intense inflammation and myelin damage, resulting in either acute or chronic neurological harm. Peripherally derived T cells, B cells, and macrophages cross the BBB and enter the CNS, releasing numerous inflammatory factors and creating localized inflammatory foci.

Advancements in technologies like single-cell sequencing and spatial transcriptomics now allow for a more detailed mapping of immune cells within the CNS, revealing their dynamic changes and functional characteristics. These developments are crucial for understanding the intricate immune microenvironment of the CNS and the immune system and are paving the way for novel treatment approaches for neurological diseases.
